# De Novo Metabolic Syndrome 1 Year after Liver Transplantation and Its Association with Mid- and Long-Term Morbidity and Mortality in Liver Recipients

**DOI:** 10.3390/jcm13061719

**Published:** 2024-03-16

**Authors:** Kinga Czarnecka, Paulina Czarnecka, Olga Tronina, Teresa Bączkowska, Magdalena Durlik

**Affiliations:** Department of Transplant Medicine, Immunology, Nephrology and Internal Diseases, Medical University of Warsaw, 59 Nowogrodzka Street, 02-006 Warsaw, Poland

**Keywords:** metabolic syndrome, liver transplantation, cardiovascular event, de novo tumours, survival, immunosuppression

## Abstract

**Background:** Metabolic syndrome (MS) constitutes an important source of cardiovascular- and cancer-related morbidity and mortality in the general population. Limited information is available on whether these findings can be directly extrapolated to liver recipients. This study aimed to investigate the impact of post-transplant MS present 1 year after liver transplantation on survival rates, risk of major cardiovascular events (CVEs), and de novo malignancies. **Methods:** Adult deceased-liver-donor recipients who underwent transplantation in our centre between 2010 and 2019 and reached at least 1 year of post-transplantation follow-up were eligible. **Results:** Of 259 enrolled patients, 20% developed post-transplant MS 1 year after the procedure. The presence of post-transplant MS at 1 year did not affect all-cause mortality (*p* = 0.144) and risk of de novo malignancies (*p* = 0.198) in liver recipients. However, it was associated with an overall and time-dependent increase in the risk of major CVEs (*p* < 0.001). MASH aetiology of liver disease, pre-existing major CVEs, and development of de novo malignancy were independent predictors of all-cause mortality in liver recipients. **Conclusions:** New onset MS exerts a wide-ranging effect on the post-transplant prognosis of liver recipients. Obtaining optimal control over all modifiable metabolic risk factors is central to improving long-term outcomes in this population.

## 1. Introduction

Metabolic disorders, cardiovascular diseases (CVDs), and cancers are among the most common complications following liver transplantation and contribute significantly to increased post-transplantation morbidity and mortality [1,2]. Metabolic syndrome (MS) reportedly develops in up to 58% of liver recipients [3,4]. CVDs affect even one-third of the liver transplantation population [4]. Similarly, a 2- to 3-fold excess of the overall risk of de novo malignancies was reported in this special population, compared to healthy controls matched for age and sex, despite the extensive oncologic work-up performed in each liver transplant candidate [5].

Data originating from the general population have demonstrated that MS constitutes an important source of cardiovascular- and cancer-related morbidity and mortality [6]. Nevertheless, it is uncertain whether these results can be directly extrapolated to the liver transplant population. Liver recipients are exposed to numerous transplant-specific risk factors, which may modulate the effect of MS on post-transplant outcomes. There is a paucity of scientific reports that provide evidence on the influence of new-onset MS on mid- and long-term prognoses in liver recipients. Those that exist suggest increased cardiovascular (CV) morbidity with no implications for survival rates and risk of de novo tumours [7,8,9,10].

MS constitutes a well-established factor fuelling the development of atherosclerotic CVDs and is known to increase the risk of major cardiovascular events (CVEs) and overall CV mortality in liver recipients [11,12]. Outside of CV-related mechanisms, the adverse impact of MS on survival is multiplied by its noxious effect on the promotion of steatosis and fibrosis of the allograft [13,14]. Additionally, MS and each of its constituents entail structural and functional changes in the kidneys, leading to the development of chronic kidney disease and favour disease progression. The underlying mechanisms of MS-associated kidney injury are complex and include, among others, insulin resistance and excessive accumulation of adipose tissue. This, in turn, promotes oxidative stress and a state of chronic inflammation, which elicit renal damage through exacerbation of pre-existing insulin resistance, activation of the renin-angiotensin-aldosterone system, alteration of endothelial function and adipocytokines imbalance [15,16]. Infection with or reactivation of oncogenic viruses, chronic antigenic stimulation leading to a cytokine-rich milieu, and chronic state of immunosuppression are the main mechanisms responsible for de novo carcinogenesis in the post-transplantation setting. Individual risk factors related to patient demographics, behavioural factors (alcohol and/or tobacco use), underlying liver disease and oncological status (malignant condition present at the time of transplantation or history of the malignant condition) are also known to contribute to carcinogenesis [17]. However, little is known about the impact of post-transplant MS on the risk of de novo malignancies in liver recipients.

Importantly, many previous investigations on this subject were restricted to short- or mid-term observations that did not account for potential confounders or collectively analysed the data of patients transplanted for non-chronic liver diseases and those with end-stage liver disease. Additionally, some studies investigating cancer-associated outcomes in liver recipients did not provide for the fact that a subset of cancer events diagnosed early in the post-transplantation period might have occurred in patients who had early-stage cancer at the time of transplantation.

MS, as a cluster of interrelated conditions, exerts multidirectional effects on a human organism and, therefore, constitutes an important determinant of post-transplant outcomes. Given the evolving profile of individuals qualified for liver transplantation and improved life expectancy following the procedure, it is crucial to further investigate and understand the extent of the impact of MS and related consequences on liver recipients in order to make a meaningful difference by mitigating the metabolic burden on post-transplant prognosis.

Therefore, this study aimed to investigate the impact of post-transplant MS present 1 year after liver transplantation on mid- and long-term survival rates, risk of major CVEs, and de novo malignancies in deceased-donor-liver recipients. Donor-, recipient- and procedure-related variables were also examined in an attempt to identify independent predictors of major CVEs and malignancies, as well as overall post-transplant mortality in the long term.

## 2. Materials and Methods

### 2.1. Study Population

This observational, retrospective study was conducted at the Department of Transplant Medicine, Immunology, Nephrology and Internal Diseases, Medical University of Warsaw. The medical records of all patients who underwent liver transplantation with a cadaveric donor due to chronic liver disease at our institution between 1 January 2010 and 31 December 2019 were reviewed. Patients who reached at least a year of post-transplant follow-up at our outpatient clinic were eligible. Patients who were under-aged at the time of transplant received organs from a living donor, or underwent re-transplantation or multi-organ transplant were ineligible. The following data were recorded from the patients’ medical files: sociodemographics (age, sex, tobacco use, alcohol abuse), aetiology of liver disease, presence of hepatocellular cancer (HCC), the model for end-stage liver disease (MELD) score, creatinine value at baseline and at 1 year post-transplant, HIV status, the occurrence of pre- and post-transplant major CVEs and malignancies, immunosuppressive protocol (at discharge and maintenance therapy at 1 year), and duration of steroid exposure. Information on patients’ anthropometric measurements and metabolic comorbidities before liver transplantation, as well as at 1, 3, 5, and 10 years after transplantation, were also extracted. The baseline weights of the liver candidates were corrected for fluid overload. The dry weight was estimated by subtracting the amount of ascites and peripheral oedema from the total body weight [18]. The characteristics of the liver donors (age, sex, weight, body mass index [BMI], waist circumference, and cause of death), along with transplant procedure-related specifics (cold and warm ischaemia time, organ sharing status), were retrieved from the National Transplant Registry. The Donor Risk Index was calculated using the equation proposed by Feng et al. [19]. MS was diagnosed according to the adapted guidelines of the International Diabetes Federation, American Heart Association, and the National Heart, Lung, and Blood Institute (Table 1) [20]. De novo MS was defined as a disorder diagnosed in the post-transplant setting.

We defined major CVEs as transient ischaemic attack, ischaemic/haemorrhagic stroke, myocardial infarction/unstable angina, or sudden cardiac death. Major CVEs that occurred as complications of intercurrent sepsis, surgical procedures, or haemorrhage were excluded. No proactive monitoring was performed in order to identify clinically silent CV conditions. De novo malignancies were defined as malignant conditions that were first diagnosed at least 6 months after liver transplantation. Cases of melanoma skin cancer were excluded from the analysis. HCC recurrence after liver transplantation was not considered an event; therefore, patients who experienced it were censored at the last follow-up date. As a general rule, all liver recipients underwent annual oncology screening with chest radiography regardless of previous smoking status and abdominal ultrasound. Mammography and cytology were ordered in female patients starting from 40 and 50 years of age, respectively. Male patients were instructed to check their prostate-specific antigen levels annually from the age of 50 years. Patients who had HCC in a native liver were additionally screened biannually with a chest-abdominopelvic CT scan with contrast for the first 3 years and once per year thereafter. This was supplemented with biannual testing of alfa-fetoprotein levels. Patients with underlying primary sclerosing cholangitis (PSC) underwent regular colonoscopy evaluations according to guidelines applicable to the general population (once every 3–5 years). Whenever PSC coexisted with inflammatory bowel disease, endoscopic examination of the lower gastrointestinal tract was ordered once per year. Patients were followed up until they were lost to follow-up, died or until the end of the study (31 December 2022). With regard to the incidence of de novo malignancies and major CVEs, liver recipients were censored at the first occurrence of malignancy and major CVEs.

Immunosuppressive treatment was instituted in accordance with the annually updated recommendations of the Polish Transplant Society [21]. Primary immunosuppression consisted of a triple-drug combination of a calcineurin inhibitor (CNI), steroid and an antimetabolic drug (mycophenolate mofetil [MMF]). Thereafter, immunosuppressive treatment was tapered according to immunological risk. Patients with an autoimmune aetiology of liver disease were maintained on a triple immunosuppressive regimen with chronic administration of low-dose steroids.

In view of the retrospective study design, the Ethics Committee approval was not required. The study protocol was submitted to the Ethics Committee of the Medical University of Warsaw for acknowledgement only (AKBE/154/2023).

### 2.2. Statistical Analysis

Continuous variables are presented as medians and ranges. Frequencies are reported for categorical variables. Based on the results of the Shapiro-Wilk test, a non-parametric Mann-Whitney U test was performed to compare continuous variables between the two groups of observations. The chi-square test or Fisher’s exact test was used to examine the relationships between categorical variables.

Proportional hazard models adjusted for sex, age at liver transplantation, metabolic dysfunction-associated steatohepatitis (MASH) aetiology of liver disease, tobacco use, and alcohol consumption were constructed to evaluate the risk of major CVEs and de novo malignancies in patients with and without new-onset MS at 1 year post-transplantation. The risk of death was evaluated using multivariate Cox regression models adjusted for sex, age at liver transplantation, MELD score and estimated glomerular filtration rate (eGFR) at 1 year as possible confounders. The Wald test was performed to verify the significance of each model. The results are presented as Kaplan-Meier curves generated from the Cox models, along with a log-rank test, hazard ratio (HR), and 95% confidence intervals. Multivariate logistic regression was used to examine the factors associated with increased risk of major CVEs, de novo tumours and all-cause mortality after liver transplantation. Initially, adjusted multivariate logistic models were examined. The best-fitted model was obtained by backward stepwise selection based on the Akaike Information Criterion. Logistic regression models were adjusted for sex, age at liver transplantation, MASH aetiology of liver disease, tobacco use, alcohol consumption for the assessment of major CVEs and de novo tumours and sex, age at liver transplantation, MELD score and eGFR at 1 year for the investigation of mortality after liver transplant.

The level of significance was set to *p* = 0.05. *p*-values indicating statistical significance are highlighted in bold.

All calculations and graphs were performed using the R statistical package version 4.0.2 (R Core Team, Vienna, Austria).

## 3. Results

**Figure 1:**
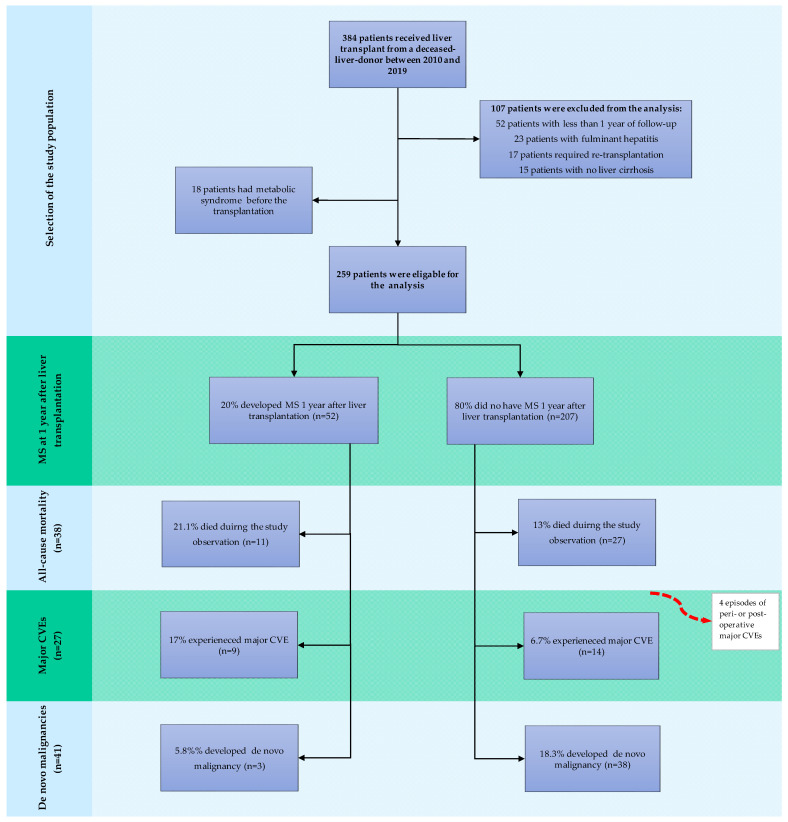
Flowchart of the study population. Abbreviations: MS, metabolic syndrome; CVE, cardiovascular event.

A total of 384 patients received liver transplants from deceased donors between 1 January 2010 and 31 December 2019, of whom 277 were initially admitted to the study (Figure 1).

The baseline characteristics, along with the transplant and donor specifics of the study population, are summarised in Table 2. Of the study participants, 18 had MS at baseline and were consequently not considered for further analysis (Figure 1). The characteristics of the remaining 259 individuals stratified by the presence of de novo MS at 1 year post-liver-transplant are presented in Table 2. Study participants were characterised by a median age of 52 (range 19–70) years and male predominance (67.1%). Hepatitis C infection and autoimmune liver diseases were the most commonly reported indications for liver transplantation in the study population, accounting for 34.7% and 24.2% of transplant procedures, respectively. MASH was a rarely reported cause of end-stage liver disease qualified for liver transplantation, accounting for 8.3% of the cases. At 1 year, a sizable proportion of liver recipients were still maintained on low-dose steroids, with only 32.5% of patients successfully converted to steroid-free regimens with tacrolimus monotherapy or CNI in combination with MMF. The median time of steroid administration was 20 (2–153) months. Metabolic disorders were rarely observed in the pre-transplant phase, with dyslipidaemia and diabetes mellitus being the dominant ones (41.5% and 28.5%, respectively). Overall, 2.9% (n = 8) of patients experienced a major CVE before the transplantation, and 2.2% (n = 6) had a history of a malignant condition. HCC in the native liver was present in 20.2% (n = 56) of the participants at transplant.

At 1 year post-transplant, 20% (n = 52) of the patients met the diagnostic criteria for new-onset MS. Individuals who developed MS after the transplant had significantly higher BMI values at baseline and were more often obese (25.2 [17.5–32.9] kg/m^2^ vs. 21.6 [15.8–30.3] kg/m^2^, *p* < 0.001; 11.5% vs. 0.5%, *p* < 0.001, for patients with and without MS respectively). Patients with MS at 1 year were also more frequently transplanted for MASH (17.3% vs. 4.3% for patients with and without MS, respectively, *p* = 0.0029). Otherwise, differences in the baseline characteristics, donors and operative variables were unremarkable between the analysed subgroups.

### 3.1. Survival

During the median follow-up of 89 months, cumulatively, 38 deaths occurred (14.7%): 11 (21.1%) in the group with post-transplant MS and 27 (13%) in the group without the condition. Infections, de novo malignancies, and CVDs were the most frequent causes of death in both groups (Table 3). COVID-19 led to fatal outcomes in one and two patients with and without new-onset MS, respectively.

**Figure 2:**
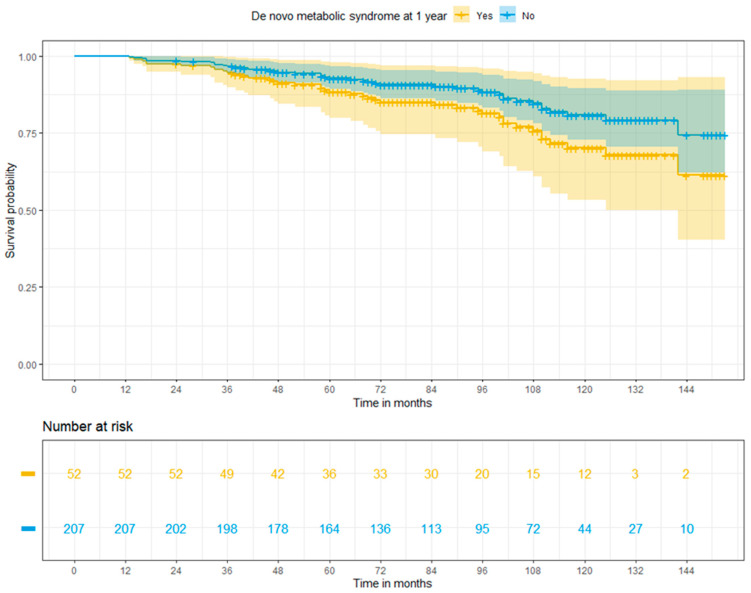
Adjusted Cox regression survival for patients with or without post-transplant metabolic syndrome at 1 year (log-rank *p* = 0.029).

Based on adjusted Cox regression analysis, MS at 1 year did not increase the overall risk of death in liver recipients (HR: 1.165; 95% CI: 0.842–3.24, *p* = 0.144). Nevertheless, Kaplan-Meier survival curves derived from the Cox regression model demonstrated a trend for inferior overall survival among patients who developed MS, with survival rates of 94.5%, 88.4%, and 70.2%, and 96.7%, 92.8%, and 80.8% for patients with and without de novo MS at 3, 5, and 10 years, respectively (*p* = 0.029) (Figure 2).

### 3.2. Major Cardiovascular Events

Overall, 27 patients (10.4%) experienced major CVEs, of which 4 (3 episodes of stroke-1 haemorrhagic, 2 ischaemic, and one event of STEMI) occurred in the direct peri- or postoperative period during the first year of observation, and therefore were excluded from further analysis. Of the remaining 23 cases, 9 occurred in patients with and 14 in patients without de novo MS (*p* = 0.0343) during a median follow-up of 83 (13–153) months. Overall, myocardial infarction/unstable angina, ischaemic/haemorrhagic stroke, and transient ischaemic attack were reported in 14, 6, and 2 patients, respectively; one patient experienced sudden cardiac death. Of the major CVEs, only one had a fatal outcome.

**Figure 3:**
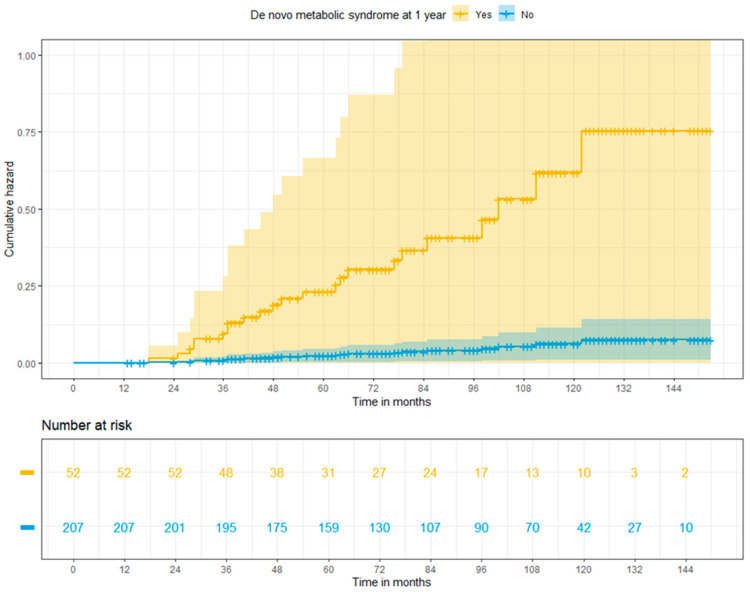
Adjusted cumulative risk of major cardiovascular events among liver recipients with or without post-transplant metabolic syndrome at 1 year (log-rank *p* < 0.001).

Patients with new onset MS showed 2.82 times higher risk of major CVEs than those who did not develop the condition (HR: 2.82; 95% CI: 1.174–6.76, *p* = 0.02). The cumulative risks of major CVEs at 3, 5 and 10 years were 9.1%, 20.6%, and 46.1% in patients with MS and 1%, 2.3%, and 6.0% in patients free from MS at 1 year (*p* < 0.001) (Figure 3).

### 3.3. De Novo Malignancies

**Figure 4:**
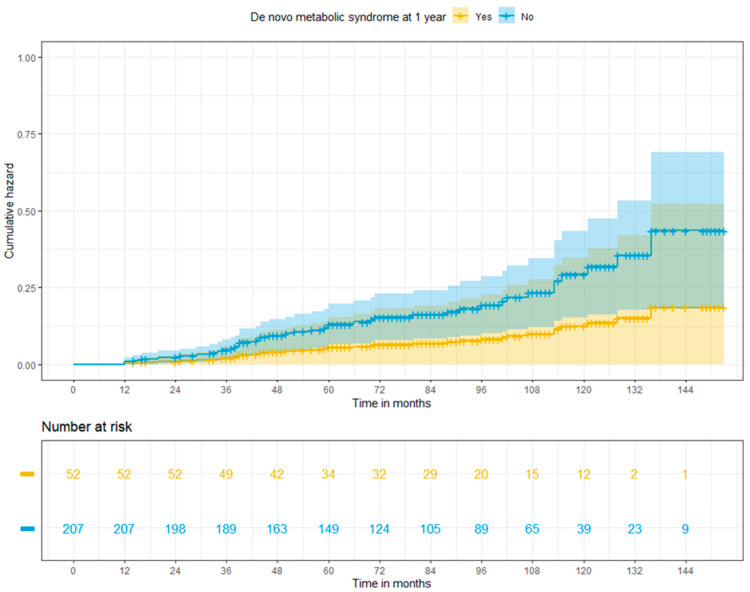
Adjusted cumulative risk of de novo tumours among patients with or without post-transplant metabolic syndrome at 1 year (log-rank *p* = 0.198).

De novo malignancies were diagnosed in 41 patients (15.8%)—3 patients with and 38 without MS (*p* = 0.0318)—within the median time from transplant of 52.4 (12–136) months. Post-transplant lymphoproliferative disorders (PTLD), head and neck cancers, and non-melanoma skin cancers were the most frequently reported de novo tumours in our population (PTLD, head and neck cancers, and non-melanoma skin cancers were reported in nine, eight, and seven patients, respectively). Overall, 24.4% (n = 10) of malignant conditions had fatal outcomes. Cumulative risks of de novo tumours at 3, 5, and 10 years were 2.0%, 3.5%, and 11.8% in patients with MS, and 4.6%, 11.3%, and 25.4% in patients free from MS at 1 year (*p* = 0.198) (Figure 4).

### 3.4. Multivariate Logistic Regression Models

In multivariate logistic regression, MASH aetiology of liver disease, major CVEs experienced prior to liver transplantation, and development of de novo tumours were independent predictors of all-cause mortality in liver recipients. Conversely, male gender was associated with increased survival probability (Table 4). However, the result was on the verge of statistical significance.

Age at liver transplantation, tobacco use, and de novo MS at 1 year were associated with increased risk of major CVEs after transplantation (Table 4).

Maintenance of steroids at 1 year post-transplantation and cyclosporine use increased the risk of de novo tumours by approximately 6.91 and 5.35 times, respectively (Table 4). The overall duration of steroid exposure did not affect the risk of post-transplant carcinogenesis (*p* = 0.799). De novo MS at 1 year was found to be protective against de novo tumours (Table 4). Risk factors commonly associated with increased risk of cancer development, such as alcohol consumption (*p* = 0.678), tobacco use (*p* = 0.948), and history of malignancy (*p* = 0.988), were not associated with increased risk of de novo tumours in liver recipients.

## 4. Discussion

This longitudinal study investigated the impact of post-transplant MS at 1 year after liver transplantation on survival rates, risk of major CVEs, and de novo malignancies in deceased-donor-liver recipients. The results demonstrate that evidence from the general population cannot be directly extrapolated to the special population of liver recipients. In our retrospective study, we found that de novo MS at 1 year post-transplant did not affect mortality figures in mid- and long-term observations after accounting for potential confounders. However, a tendency for poorer survival among patients with MS was demonstrated. Post-transplant MS was associated with an overall and time-dependent increase in the risk of major CVEs, while the development of de novo malignancies remained unaffected. This indicates that transplant-specific factors significantly modulate the effect of MS on post-transplant outcomes and outweigh the impact of traditional risk factors in terms of carcinogenesis.

Our study cohort had excellent survival rates in both subgroups. Our results surpassed values reported in other studies, which documented 83% survival at 5 years and 71% survival at 10 years [22]. This discrepancy may be explained by our study design, which eliminated many subjects affected by well-documented factors for poor post-transplantation survival.

Our results demonstrated that pre-existing CVEs were the most impactful determinants of all-cause mortality. Accordingly, in order to be considered for the transplant procedure, liver transplant candidates undergo detailed cardiac risk stratification. In view of the great heterogeneity of cirrhosis-related cardiac conditions, blunted cardiac response to stress, progression of underlying cardiac diseases over time, lack of standardised screening protocols, and metabolic pandemics among the general population, an accurate cardiac evaluation remains a challenge in the evolving landscape of liver transplantation [23,24]. To further complicate matters, non-invasive functional assessment has been evidenced to produce a relatively low capacity in detecting coronary artery disease and low accuracy in predicting the risk of major CVEs in the peri- or postoperative setting [25,26,27]. Finally, it is also recognised that patients may be hesitant to report cardiac-associated symptoms for fear of being rejected from this life-saving procedure. Therefore, some of the cardiac risk factors/conditions remain unmitigated or even unrecognised. Noteworthy, due to the lack of transplant-specific recommendations for CV surveillance in liver recipients, guidelines for the general population are applied [28]. Therefore, it appears prudent to implement additional post-transplantation CV screening tailored to the individual risk profile of each liver recipient in order to optimise survival statistics in this group of patients.

Although no statistically significant differences were noted between kidney function in patients with and without MS 1 year after transplantation, patients in the former group fared worse in terms of renal parameters. Given the underlying pathophysiological mechanisms of MS-induced renal injury, it is likely that these initially insignificant findings deteriorated over the course of follow-up and along with the increasing number of MS components ultimately contributed to increased CV morbidity and mortality secondary to accelerated loss of renal function [29].

**Figure 5:**
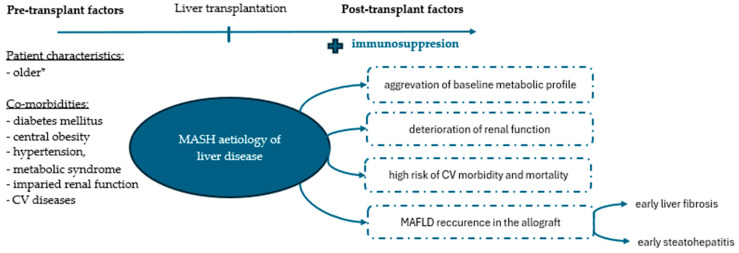
Metabolic profile of liver recipients with underlying metabolism-associated steatohepatitis (MASH). *As compared to patients transplanted for other indications. Abbreviations: CV, cardiovascular; MAFLD, metabolic dysfunction-associated liver disease.

The post-transplantation prognosis of patients with MASH is a subject of ongoing debate. Some studies have reported comparable patient and graft survival rates between patients transplanted for MASH and non-MASH-related causes [30,31,32,33,34]. Others have contradicted this statement, demonstrating increased mortality, predominantly of CV or cerebrovascular aetiology, in patients with MASH [35,36,37,38]. The latter finding is supported by our results and likely attributable to the metabolic profile of patients with underlying MASH (Figure 5) [36,38]. The risk of poor post-transplantation outcomes in patients with MASH is further amplified by the frequent coexistence of sarcopenia and frailty [39]. It is projected that up to 62% of patients with MASH suffer concomitant sarcopenia that does not improve after liver transplantation [40,41]. Instead, it often persists and likely worsens in the long-term observation [41]. Furthermore, MASH and sarcopenia are uniquely intermeshed [42]. Both entities have been evidenced to share many pathophysiological pathways leading to increased insulin resistance and inflammation [39,42]. Accordingly, their coexistence produces unfavourable synergistic effects [42]. Finally, frailty reportedly affects approximately 50% of patients with end-stage liver disease due to underlying MASH, which considerably exceeds projections for their non-MASH peers [43]. These dismal statistics are likely attributed to their multimorbidity and are associated with suboptimal prognosis for post-transplant recovery. As demonstrated by Lai et al., less than 40% of patients achieve robustness after the surgery [44]. All of those, combined with repeated hospitalisations, long-term immunosuppressive treatment, older age, frequent physical inactivity, poor nutritional habits, and post-transplantation gain in weight, likely facilitate conditions for the development of sarcopenic obesity rather than the desired rebuild of muscle mass and post-cirrhosis convalescence. To complicate matters even further, to date, no unified diagnostic criteria exist to accurately address sarcopenia [45,46]. The lack of appropriate animal models impedes further investigations and a better understanding of the underlying pathomechanisms [45]. Thus far, early initiation of physical activity and dietary and nutritional management combined with optimal control of metabolic dysregulation remain the cornerstone in the combat against frailty and sarcopenia [46].

One may find it surprising that MASH, but not post-transplant MS, was associated with inferior survival probability in our study. This may be explained by several factors. By definition, MS is associated with hepatic steatosis, which per se does not translate into an increased risk of post-transplant mortality [47,48]. Importantly, steatosis of the allograft can develop de novo or result from the recurrence of the underlying liver disease. Previous studies demonstrated that recurrent metabolic-associated fatty liver disease (MAFLD) is more prevalent in patients transplanted for MASH and confers significant clinical and prognostic implications for liver recipients as compared to de novo cases [14,49]. The recurrent nature of the disease was associated with the early development of severe fibrosis and steatohepatitis in the transplanted liver [14,49]. Vallin et al. also suggested that cases of recurrent MAFLD constitute an irreversible disease [49]. Accordingly, many studies point towards the recurrence of MASH and liver fibrosis as an important source of post-transplant deaths [47].

To date, there is no established data supporting gender outcome disparities in the liver transplant setting. The limited data available indicate poorer short-term survival and favourable long-term outcomes in women compared to men [50,51]. Our results are at variance with these preliminary reports. In our cohort, men presented approximately a 3-fold lower risk of all-cause mortality. Potential confounding factors such as age, renal function, and MELD score were accounted for. Furthermore, recipient gender was not associated with increased CV (*p* = 0.199) and cancer risk (*p* = 0.161). Considering that more than half of the female participants were over the age of 52, our results may be partially attributed to hormonal changes associated with menopause. The menopausal transition is known to promote significant weight gain and abdominal fat accumulation—well-established risk factors for MASH, CVEs, and de novo malignancies [52]. Nevertheless, this finding should be approached with caution as our study design was not empowered to accurately investigate the influence of gender outcome disparities on post-transplantation prognosis.

Post-transplant MS was previously found to confer approximately four times greater risk of CVEs, with incidence rates ranging from 10–20% at 3–5 years [4,53,54,55]. Our study partially replicates these findings. Notably, despite the longer follow-up period, the overall incidence of major CVEs in our study was comparable to that reported in previous research. However, our results might have been underestimated owing to the study design and lack of routine CV screening. On the other hand, with very few exceptions, liver recipients in our transplant centre were maintained on tacrolimus-based immunosuppression protocols, which have been associated with lower CV risk and fewer metabolic implications than cyclosporine- and non-calcineurin-based regimens [4,56]. Notably, the incidence of major CVEs reported in our study did not translate into increased CV mortality. This is likely due to better pre-transplant CV risk stratification strategies, diligent aftercare, advances in the management of identified CV risks and diseases, and improvements in non-invasive cardiological interventions. The development of major CVEs was also independently predicted by traditional CV risk factors, such as older age at transplantation and tobacco use.

PTLD, non-melanoma skin cancers, and head and neck cancers are the most common types of malignancies reported in liver recipients [17]. This finding is replicated in the present study. Reportedly, the incidence of de novo malignancies varies from 2.6% to 26%, depending on the study design, population under investigation, era of transplantation and time of follow-up [17,57]. The incidence of de novo carcinogenesis in our study was comparable to that range, accounting for 15.8%. Noteworthy, despite the routine surveillance strategy in place, de novo tumours constituted the second most frequently reported cause of death in our population. Accordingly, previous studies support our results and list malignancies among the leading causes of mid- and long-term mortality in the liver transplant population, with a reported constant upward trend [1,17]. Given the ageing population, longer exposure to immunosuppression, prevalent metabolic comorbidities, and frequent physical inactivity of organ recipients, these increasing trends may be anticipated.

Our results identified post-transplant MS as a potential protective factor against de novo carcinogenesis in liver recipients. Considering the well-documented relationship between MS and several cancers, one may find our results surprising. After a thorough analysis of the data, we concluded that the reason for this could be multifactorial and related to the pattern of cancer occurrence, baseline characteristics of the study population, and application of a screening program. Previous reports have linked MS with an increased risk of gastrointestinal cancer in both sexes, bladder cancer in men, and malignancies of the reproductive system in women [58]. Of note, more than half of the malignancy-related events reported in our study were not traditionally linked with metabolic status [17]. Additionally, our screening program might have resulted in an effective prophylactic strategy for MS-related malignancies but failed to address the increased risk of PTLD and skin and head and neck cancers. Furthermore, it is suggested that in patients with MS, intercurrent obesity outweighs the effect of metabolic health on cancer risk [59]. As a result, MS, as a cluster of interlinked yet independent conditions that do not equally contribute to carcinogenesis, may not be the point of reference for assessing the risk of malignancies. Based on these arguments, we cannot conclude that post-transplant MS decreases the risk of de novo tumours in liver recipients.

Long-term exposure to immunosuppressive agents has been long-indicated as the primary mechanism responsible for the increased risk of carcinogenesis in solid-organ recipients. This was substantiated by our results, which showed that prolonged, deep suppression of the immune system poses a significant risk for de novo tumours. Study participants who were uninterruptedly exposed to steroids up until 1 year after the transplant were almost seven times more likely to develop de novo malignancy. Our findings are also supported by emerging investigations in the general population, which demonstrated that prolonged systemic exposure to steroids results in an increased risk of cancer in a dose-dependent manner [60,61].

Previous publications consistently reported the contribution of CNIs to carcinogenesis outside of their immunosuppressive potential, exerted primarily through the promotion of transforming growth factor (TGF)-β expression [57]. Accordingly, we determined that patients maintained on cyclosporine had an increased risk of malignancies. Importantly, we found such an association only for cyclosporine but not for tacrolimus, another drug from the CNI group, despite a largely shared mechanism of action. It is hypothesised that, compared to tacrolimus, cyclosporine produces greater suppression of the immune system and induces higher levels of TGF-β. Consequently, it enhances proliferation and diminishes the differentiation and apoptosis of cancer cells to a greater extent. In vivo studies have also documented the impact of cyclosporine on tumour progression and angiogenesis [62,63]. Our findings provide additional evidence supporting early steroid withdrawal from the immunosuppressive regimen whenever clinically indicated and weighed in favour of tacrolimus-based immunosuppression protocols.

Our study was limited by its retrospective and unicentric design. Due to the lack of waist circumference measurements, BMI was substituted as a surrogate indicator for abdominal obesity to diagnose MS. Furthermore, the incidence of de novo malignancies and major CVEs reported in our study might have been underestimated, as participants were censored at the first occurrence of the event. Additionally, we were unable to detect clinically silent episodes of major CVEs as no routine CV screening was instituted. We also acknowledge that some skin lesions may have been removed or ablated without histopathological examination, which precluded their inclusion in our analysis. Moreover, our study population consisted only of Caucasian adults, which may limit the generalizability of our results worldwide. Finally, we did not gauge the level of compliance with our screening protocol.

## 5. Conclusions

Considering the above-mentioned facts, post-transplant MS constitutes a mounting challenge for liver recipients as an important determinant of poor post-transplant prognosis. Despite the lack of statistical significance in the adjusted Cox regression model, a trend for poorer survival was demonstrated in patients who developed MS at 1 year post-transplant compared to those who did not. Post-transplant MS was also associated with an overall and time-dependent increase in the risk of major CVEs, whereas no such association was found for the risk of de novo malignancies. Increased risk of carcinogenesis was associated with transplant-specific risk factors such as prolonged steroid use and cyclosporine-based maintenance immunosuppressive protocols. Our results weigh in favour of tacrolimus-based immunosuppression to mitigate cardiac- and cancer-related morbidity compared to cyclosporine-based regimens. Considering the wide-ranging effects of MS on post-transplant prognosis, it is of paramount importance to put emphasis on the prevention, early recognition, and adequate management of MS and all its modifiable constituents in order to improve the late outcomes of liver recipients. To achieve this goal, the joint efforts of all healthcare professionals caring for liver recipients are the key to success. Transplant specialists should concentrate on appropriate selection and subsequent modification of immunosuppressive therapy to reduce the risk of iatrogenic side effects. Optimal and regular monitoring of blood pressure values, diabetes mellitus and body weight parameters should be carried out in close cooperation with transplant centres, diabetologists, cardiologists and primary care physicians. All professionals should complement each other in educational efforts regarding daily activities/habits and dietary measures. Furthermore, it appears crucial to broaden the awareness of the transplant-specific risk factors and associated consequences among all healthcare professionals in order to support patients through consistent messaging and approach across all specialists. Liver recipients would also benefit from combined efforts to gauge the level of compliance with surveillance protocols. 

## Figures and Tables

**Table 1:** Adapted guidelines of the International Diabetes Federation, American Heart Association, and the National Heart, Lung, and Blood Institute used to diagnose metabolic syndrome.

Simultaneous Coexistence of at Least Three out of the Five Following Factors Resulted in MS Diagnosis:
BMI ≥ 30 kg/m^2^ Triglycerides ≥ 150 mg/dL (1.7 mmol/L) or drug treatment for elevated triglycerides HDL cholesterol of ≤40 mg/dL (1.0 mmol/L) in males; ≤50 mg/dL (1.3 mmol/L) in females or specific treatment for this lipid abnormality Systolic blood pressure ≥ 130 mmHg; diastolic blood pressure ≥ 85 mmHg, or hypotensive pharmacological treatment in a patient with a medical history of hypertension Fasting glucose ≥ 100 mg/dL (5.5 mmol/L) or pharmacological management of previously diagnosed diabetes mellitus

Abbreviations: MS, metabolic syndrome; BMI, body mass index; HDL, high-density lipoprotein.

**Table 2:** Characteristics of the study population stratified by presence of post-transplant metabolic syndrome at 1 year.

Variable	Overall (n = 277)	PTMS (n = 52)	None PTMS (n = 207)	Test	*p*-Value *
**Characteristics of liver recipients**					
Male sex	67.1% (n = 186)	73.1% (n = 38)	64.7% (n = 134)	chi-squared	0.3298
Age at liver transplantation [years]	52 (19–70)	54 (36–67)	51 (19–70)	Mann-Whitney U	0.2059
MELD score	16 (7–34)	16 (7–30)	16 (7–34)	Mann-Whitney U	0.5878
**Pre-transplant BMI [kg/m^2^]**	22.1 (15.8–32.9)	25.2 (17.5–32.9)	21.6 (15.8–30.3)	Mann-Whitney U	**<0.001**
**Pre-transplant obesity**	3.2% (n = 9)	11.5% (n = 6)	0.5% (n = 1)	Fisher	**<0.001**
Pre-transplant diabetes mellitus	28.5% (n = 79)	34.6% (n = 18)	21.7% (n = 45)	chi-squared	0.0794
Pre-transplant hypertension	14.4% (n = 40)	17.3% (n = 9)	9.7% (n = 20)	chi-squared	0.1878
Pre-transplant dyslipidaemia	41.5% (n = 115)	48.1% (n = 25)	34.8% (n = 72)	chi-squared	0.1073
Pre-transplant metabolic syndrome	6.5% (n = 18)	0% (n = 0)	0% (n = 0)	Fisher	1
Pre-transplant creatinine [mg/dl]	0.9 (0.4–5.27)	0.92 (0.4–4.6)	0.87 (0.4–5.27)	Mann-Whitney U	0.1178
Tobacco use	9.7% (n = 27)	7.7% (n = 4)	10.6% (n = 22)	Fisher	0.796
Alcohol consumption	25.3% (n = 70)	34.6% (n = 18)	24.6% (n = 51)	chi-squared	0.2007
**Indication for liver transplantation**					
HCV	34.7% (n = 96)	28.8% (n = 15)	36.7% (n = 76)	chi-squared	0.368
PSC, PBC, AIH	24.2% (n = 67)	15.4% (n = 8)	25.6% (n = 53)	chi-squared	0.1707
ALD	18.1% (n = 50)	25% (n = 13)	17.9% (n = 37)	chi-squared	0.3334
HBV	10.5% (n = 29)	11.5% (n = 6)	10.6% (n = 22)	chi-squared	1
**MASH**	8.3% (n= 23)	17.3% (n = 9)	4.3% (n = 9)	chi-squared	**0.0029**
Other	4.3% (n = 12)	1.9% (n = 1)	4.8% (n = 10)	Fisher	0.6992
Concomitant HCC	20.2% (n = 56)	19.2% (n = 10)	19.3% (n = 40)	chi-squared	1
**Donor characteristics**					
Male sex	59.2% (n = 164)	65.4% (n = 34)	60.4% (n = 125)	chi-squared	0.6153
Age [years]	41 (10–75)	39.5 (19–66)	41 (28–50.5)	Mann-Whitney U	0.7821
BMI [kg/m^2^]	24.7 (15.6–41.6)	24.6 (18.8–41.6)	24.6 (15.6–34.7)	Mann-Whitney U	0.8246
Waist circumference [cm]	85 (64–120)	86.5 (64–116)	84 (64–120)	Mann-Whitney U	0.5667
**Cause of donor death**					
Trauma	38.3% (n = 106)	32.7% (n = 17)	40.6% (n = 84)	chi-squared	0.377
Cerebrovascular accident	50.5% (n = 140)	55.8% (n = 29)	48.8% (n = 101)	chi-squared	0.4566
Anoxia	6.9% (n = 19)	9.6% (n = 5)	6.8% (n = 14)	Fisher	0.5505
Other	4.3% (n = 12)	1.9% (n = 1)	3.9% (n = 8)	Fisher	0.6921
**Operative characteristics**					
Warm ischemia time [min]	40 (23–71)	39 (28–70)	40 (23–71)	Mann-Whitney U	0.4636
Cold ischemia time [min]	390 (126–900)	397.5 (225–725)	385 (126–780)	Mann-Whitney U	0.6111
Donor Risk Index	1.48 (0.92–2.44)	1.41 (1.11–2.26)	1.48 (0.92–2.44)	Mann-Whitney U	0.6612
**Immunosuppression at 1 year**					
triple therapy with steroids + MMF+ CNI/EVR	42.6% (n = 118)	46.2% (n = 24)	40.6% (n = 84)	chi-squared	0.5677
dual therapy with steroids + CNI/EVR	22.4% (n = 62)	23.1% (n = 12)	22.7% (n = 47)	chi-squared	1
dual therapy with MMF + CNI	15.5% (n = 43)	19.2% (n = 10)	15% (n = 31)	chi-squared	0.5899
monotherapy with TAC	15.5% (n = 43)	11.5% (n = 6)	16.4% (n = 34)	chi-squared	0.5111
other	4% (n = 11)	0% (n = 0)	5.3% (n = 11)	Fisher	0.1278
**Immunosuppressive drugs at 1 year**					
Steroids	67.5% (n = 187)	69.2% (n = 36)	66.7% (n = 138)	chi-squared	0.8518
TAC	92.8% (n = 257)	98.1% (n = 51)	91.3% (n = 189)	Fisher	0.1353
MMF	58.1% (n = 161)	65.4% (n = 34)	55.6% (n = 115)	chi-squared	0.2606
Cyclosporine	6.1% (n = 17)	1.9% (n = 1)	7.2% (n = 15)	Fisher	0.2072
EVR	3.6% (n = 10)	0% (n = 0)	4.8% (n = 10)	Fisher	0.2197
Azathioprine	1.4% (n = 4)	0% (n = 0)	1.9% (n = 4)	Fisher	0.5864
**Duration of steroid exposure [months]**	20 (2–153)	19 (2–151)	20 (2–153)	Mann-Whitney U	0.7139
**Duration of follow-up [months]**	89 (13–153)	85 (28–151)	88 (13–153)	Mann-Whitney U	0.3622

Continuous variables are presented as medians (ranges). * *p*-values were calculated by comparing patients with PTMS with those without PTMS. Abbreviations: PTMS, post-transplant metabolic syndrome; MELD, model for end-stage liver disease; BMI, body mass index; HCV, hepatitis C virus; PBC, primary biliary cirrhosis; AIH, autoimmune hepatitis; PSC, primary sclerosing cholangitis; ALD, alcoholic liver disease; HBV, hepatitis B virus; MASH, metabolic dysfunction-associated steatohepatitis; HCC, hepatocellular carcinoma; CNI, calcineurin inhibitor; TAC, tacrolimus; MMF, mycophenolate mofetil; EVR, everolimus.

**Table 3:** Causes of death stratified by the presence of post-transplant metabolic syndrome.

Cause of Death	PTMS (n = 52)	no PTMS (n = 207)
totals deaths	11 (21.15%)	27 (12.98%)
cardiovascular conditions	1 (9%)	3 (11.1%)
infections (including COVID-19)	5 (45.5%)	9 (33.3%%)
malignant disease	1 (9%)	9 (33.3%)
miscellaneous *	4 (36.7%)	6 (22.2%)

* Two cases of kidney failure, one case of recurrent liver cirrhosis, one case of accident, two cases of graft failure, two cases of hepatocellular cancer recurrence, one case of suicide, and one case of biliary complications. Abbreviations: PTMS: post-transplant metabolic syndrome.

**Table 4:** Adjusted multivariate logistic regression for factors associated with overall survival, major cardiovascular events, and de novo tumours.

Variable	Estimate	*p*-Value	OR	LCI	UCI
**Survival**					
MASH aetiology of liver disease	1.548	**0.012**	4.700	1.386	16.071
Pre-transplant cardiovascular event	2.919	**0.002**	18.514	3.196	156.375
De novo tumour	1.363	**0.004**	3.908	1.524	9.956
Male gender	−1.127	**0.049**	0.324	0.105	1.015
**Major CVEs**					
Age at liver transplantation	0.127	**<0.001**	1.135	1.061	1.229
Tobacco use	2.216	**0.006**	9.169	1.948	48.270
Post-transplant metabolic syndrome at 1 year	1.409	**0.033**	4.091	1.141	15.694
**De novo malignancies**					
Steroids use at 1 year post-transplant	1.933	**0.036**	6.908	1.144	46.701
Cyclosporine use at maintenance	1.677	**0.009**	5.349	1.483	19.267
Post-transplant metabolic syndrome at 1 year	−1.545	**0.021**	0.213	0.046	0.691

Abbreviations: MASH, metabolic dysfunction associated steatohepatitis; CVEs, cardiovascular events.

## Data Availability

Data supporting the results reported in the article can be found under the following https://doi.org/10.17632/jzxsj7kr7m.1.
